# Supplementary material for: Inactivation of KCNQ1 potassium channels reveals dynamic coupling between voltage sensing and pore opening
Source: Nat Commun. 2017 Nov 23;8:1730. doi: 10.1038/s41467-017-01911-8 (PMC5700111; doi:10.1038/s41467-017-01911-8)
Supplement: Supplementary file 1 — Supplementary Information [file 41467_2017_1911_MOESM1_ESM.doc]

**
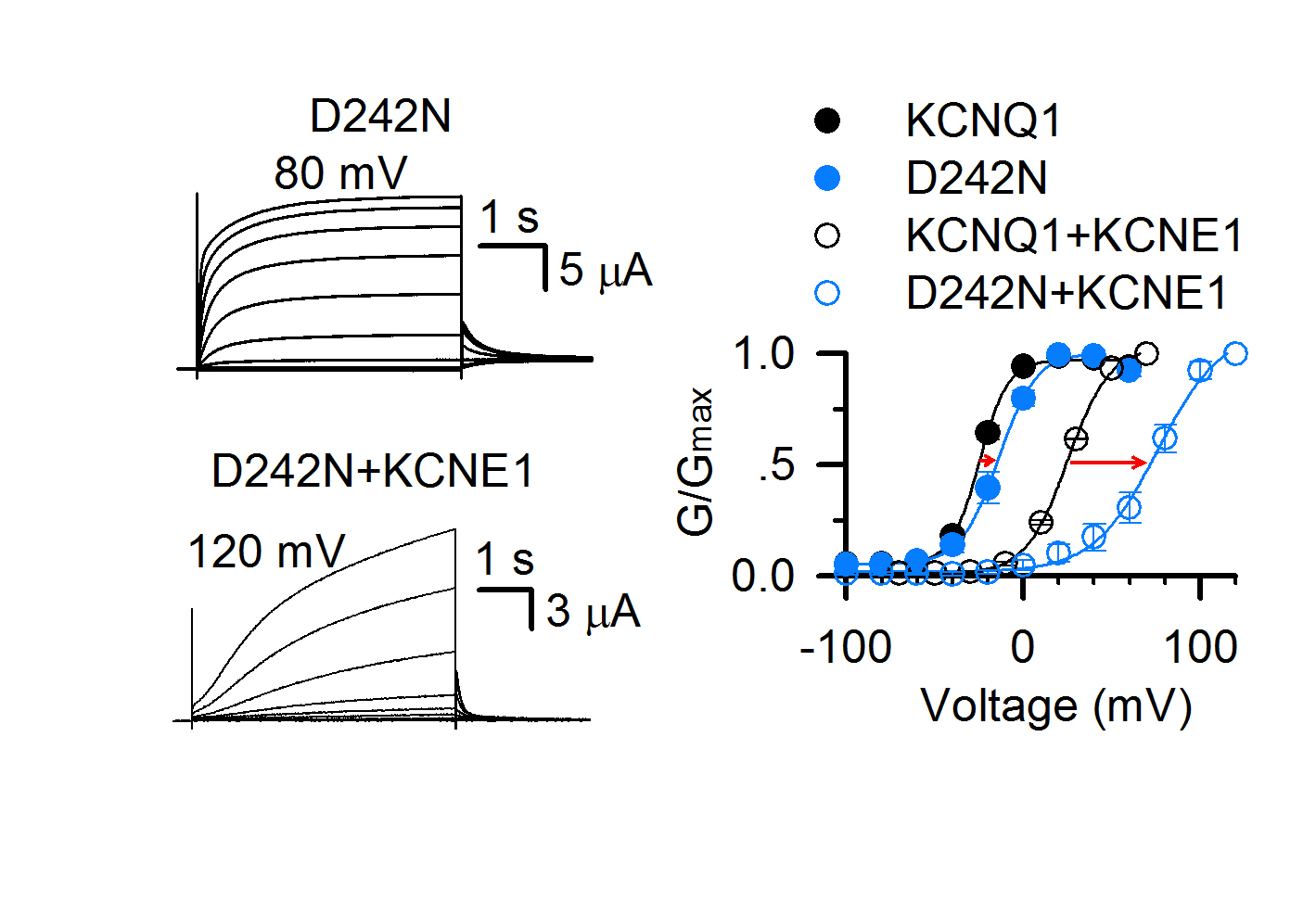
**

**Supplementary Figure 1. Voltage dependent activation of D242N and D242N+KCNE1.** Left, representative D242N and D242N+KCNE1 activation currents. Right, G–V relationships of KCNQ1, KCNQ1+KCNE1, D242N, and D242N+KCNE1. n≥4. Error bars are the SEM.


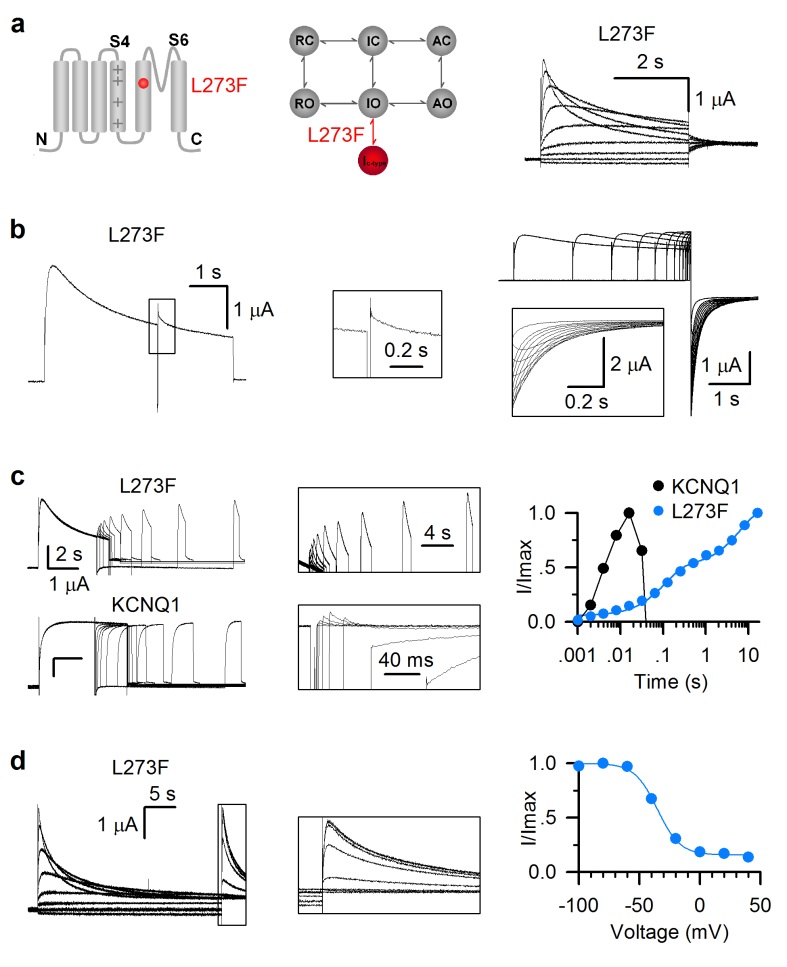


**Supplementary Figure 2. Long QT mutation L273F induces C type inactivation, without affecting the IO↔AO transition. (a)** Left, scheme to show that L273F induces a C-type inactivated state (red circle) to the channel. Right, the L273F currents recorded in ND96 solution. **(b)** Left, representative KCNQ1 fast decaying current recorded in ND96 solution from a triple pulse protocol. The hyperpolarizing pulse was for 20 ms at -120 mV, and the depolarizing pulse was to +40 mV. Middle, the fast decaying current with expanded current and time scales. Right, time dependence of L273F hook currents recorded in high potassium (100 mM K^+^) solution. The pre-pulses were +40 mV with time durations ranging between 0.02 - 4.355 s, and the test pulse was 2 s long at - 120 mV. The inset shows the hook in tail currents with an expanded time scale. **(c)** Representative currents of recovery from inactivations of L273F (top) and KCNQ1 (bottom). The test pulse was +40 mV, and the recovery voltage was -120 mV with 0.001 - 16 s time durations. The insets in the middle panel were enlarged recovery currents. The scale bars were shown as indicated. Right, the recovery curves of KCNQ1 (black) and L273F (blue). The L273F curve was fitted with a double exponential function. **(d)** Steady state inactivation currents of L273F. Currents were recorded with a 30 s pre-pulse at voltage range from -100 to +40 mV, and the test pulse was 4 s at +40 mV. The right panel showed the steady state inactivation curve of L273F.

**Supplementary table 1. All primer sequences used in this study.**

| Mutations | Primer sequences |
| --- | --- |
| C214A | pB: cttggaTcccacCGCgaggaccacca  pC: cGCGgtgggAtccaaggggcaggtg |
| G219C | pB: cctgaCacttggaTcccacCGC  pC: ggAtccaagtGtcaggtgtttgccacg |
| C331A | pB: gacagagaaTGCggaggcgatggtcttc  pC: ctccGCAttctctgtctttgccatc |
| F351A | pB: ggcaGCGcccgagccaagaatcc  pC: gctcgggCGCtgccctgaaggtgcag |
| S338F | pB: gaagAATatTgcaaagacagagaag  pC: gtctttgcAatATTcttctttgcgctccc |
| E160R | pB: cgatTCTcatccagaagagagt  pC: ctcttctggatgAGAatcgtgctggtggtgttct |
| R231E | pB: caggaaTTCgatgcccctgatggc  pC: gggcatcGAAttcctgcagatcctgag |
| R237E | pB: gcatcTCcaggatctgcaggaag  pC: cagatcctgGAgatgctacacgtcgac |
| D242N | pB: ggcggtTgacgtgtagcatcctc  pC: ctacacgtcAaccgccagggagg |
| L273F | pB: gaagatgaATcccaggaagccgatg  pC: cctgggATtcatcttctcctcgtac |
